# "Solving vessel caliber mismatch in microvascular anastomosis: A comprehensive review, novel techniques, and a surgical guide for optimal outcomes"

**DOI:** 10.1016/j.jham.2024.100179

**Published:** 2024-11-20

**Authors:** Riccardo Nocini, Valentina Pinto, Luca Contu, Giorgio De Santis, Marco Pignatti

**Affiliations:** a Ear, Nose and Throat, Department of Surgical Sciences, Dentistry, Gynaecology and Paediatrics, University of Verona, Verona, Italy; b Plastic Surgery, Policlinico di Modena, University of Modena and Reggio Emilia, Italy; c Plastic Surgery, IRCCS AOU di Bologna, Policlinico di Sant'Orsola, Bologna, Italy; d Department of Medical and Surgical Sciences (DIMEC), University of Bologna, Bologna, Italy

**Keywords:** Microsurgery, Vascular anastomosis, Caliber mismatch, Caliber discrepancy, Surgical technique, Guide, Data availability statement: all articles reviewed are available online, Funding statement: no founding, Conflict of interest disclosure: no conflict of interest

## Abstract

Caliber mismatch in microvascular anastomosis can significantly increase procedural difficulty and elevate the risk of thrombosis. A comprehensive literature search in PubMed, Scopus, Web of Science, and Google Scholar was conducted to identify articles addressing surgical techniques for overcoming caliber mismatch in microvascular anastomosis. Various techniques described in the literature were found: modifications of end-to-end anastomosis, the use of end-to-side anastomosis, the application of vessel grafts and the use of vessel couplers. In this review, we critically analyze these techniques and introduce additional technical variations. We discuss the options and express our preferred choice of methods based on specific clinical scenarios: if an alternative vessel (either new or isolated further away) is not found, the severity of the mismatch guides the choice. When less then 1/3 our choice is for vessel dilation and oblique cut of the smaller vessel end (if necessary with the adjunct of a titanium small Ligaclip in an oblique fashion to avoid a cul-de-sac). If caliber mismatch is around or over 1/3, we would prefer an end to side anastomosis.

## 1 Introduction

Microvascular anastomosis is a fundamental technique in reconstructive surgery, allowing for the reestablishment of blood flow between two vessels. This procedure is critical in various clinical applications, including organ transplantation, trauma vascular repair, and free tissue transfers. However, performing anastomosis between vessels of different sizes presents significant challenges that can impact the success of the surgery. The disparity in vessel diameter can lead to complications such as stenosis and thrombosis, compromising blood flow.^1^

Different surgical techniques have been proposed to perform a microvascular anastomosis between vessels of different caliber to optimize patency.

This review paper aims to provide a comprehensive overview of those different techniques. By examining the indications, advantages, and limitations of these methods, we seek to offer valuable insights for surgeons in selecting the most appropriate technique for specific scenarios. The techniques reviewed include end-to-end anastomosis (ETE) with different options, end-to-side anastomosis (ETS), interpositional grafts, and the use of microvascular couplers.

We also present our method to choose the most appropriate surgical technique to overcome vessel caliber discrepancy depending on the specific clinical scenarios.

## 2 Materials and methods

A comprehensive literature search was conducted to identify articles addressing surgical techniques for overcoming caliber mismatch in microvascular anastomosis. The following electronic databases were utilized: PubMed, Scopus, Web of Science, and Google Scholar. The search strategy incorporated a combination of keywords and MeSH terms including "microvascular anastomosis," "caliber mismatch," "surgical techniques," "vessel size discrepancy," and "reconstructive surgery."

### 2.1 Inclusion criteria

1. Articles published in peer-reviewed journals.
2. Studies that specifically address surgical techniques for microvascular anastomosis with caliber mismatch.
3. Research articles, reviews, clinical trials, and case studies.
4. Publications in English.
5. Articles published from 1990 to 2023 to ensure a comprehensive and up-to-date review.

### 2.2 Exclusion criteria

1. Studies focusing solely on non-surgical techniques.
2. Articles not addressing caliber mismatch in microvascular anastomosis.
3. Non-English publications.
4. Conference abstracts, letters to the editor, and opinion pieces without substantial data.

Data extraction was performed independently by two reviewers. The extracted data included.

• Authors and publication year.
• Study design (e.g., clinical trial, retrospective study, review).
• Type of surgical technique discussed.
• Outcomes measured (e.g., patency rates, complication rates, functional outcomes) (if available)
• Key findings and conclusions.

Discrepancies between reviewers were resolved through discussion and consensus. In cases where consensus could not be reached, a third reviewer was consulted.

Due to the heterogeneity of study designs and reported outcomes, a primarily descriptive approach was utilized.

The choice of the most suitable technique to overcome a specific caliber mismatch is not easy. Experience of the microsurgeon often has a crucial role. We share our opinion on how to choose the most suitable surgical technique.

### 2.3 Ethical considerations

As this study involved a review of existing literature, ethical approval was not required. However, all included studies were evaluated for ethical considerations, ensuring that they adhered to the ethical standards of their respective institutions and journals.

## 3 Results

The literature review produced 58 articles related to the subject.

After exclusion of non-relevant papers, we identified 46 eligible articles^1^, ^2^, ^3^, ^4^, ^5^, ^6^, ^7^, ^8^, ^9^, ^10^, ^11^, ^12^, ^13^, ^14^, ^15^, ^16^, ^17^, ^18^, ^19^, ^20^, ^21^, ^22^, ^23^, ^24^, ^25^, ^26^, ^27^, ^28^, ^29^, ^30^, ^31^, ^32^, ^33^, ^34^, ^35^, ^36^, ^37^, ^38^, ^39^, ^40^, ^41^, ^42^, ^43^, ^44^, ^45^, ^46^ and performed a further choice of the articles describing specific surgical techniques used to solve a caliber mismatch in microvascular anastomosis.

Those 25 articles are are reported in detail in Table 1.^4^, ^5^, ^6^, ^7^, ^8^, ^9^, ^10^, ^11^, ^12^, ^13^, ^14^, ^15^, ^16^, ^17^, ^18^, ^19^, ^20^, ^21^, ^22^, ^23^, ^24^, ^25^, ^26^, ^27^, ^28^

**Table 1:** Literature review details.

Study	Year	Journal	Technique	Model
B. J. Brener et al.^4^	1974	Surg Gynecol Obstet	Oblique Section	/
C. Lauritzen^5^	1978	Scand J Plast Reconstr Surg	Sleeve Anastomoses Invaginating Technique	Animal
T. Harashina et al.^6^	1980	Plast Reconstr Surg.	Fish-Mouth Incision	Animal
T. Harashina et al.^7^	1983	Microsurgery	Wedge Excision	Animal
A. D. Ryan et al.^8^	1988	Plast Reconstr Surg.	Interposition Vein Graft	Animal
G. J. Gumley et al.^9^	1989	Br J Plast Surg.	Thin-Wall Interposition Vein Graft	Animal
J. J. Monsivais^10^	1990	Microsurgery	Interposition Vein Graft	Animal
Z. F. Xiu et al.^11^	1993	Br J Plast Surg.	Unequal Bite Technique	Human
C. Y. Ahn et al.^12^	1994	Ann Plast Surg.	Interposition Vein Graft	Animal
K. Ueda et al.^13^	1994	J Reconstr Microsurg.	Distal Tapering	Human
E Yüksel et al.^14^	1999	J Reconstr Microsurg.	Interposition Arterial Graft Previous Dilatation with PTCA Catheter	Animal
J. A. de la Peña-Salcedo et al.^15^	2000	Microsurgery	Experimental Microvascular Sleeve Anastomosis	Animal
S. K. Sullivan et al.^16^	2003	J Reconstr Microsurg.	Microvascular Venous Coupler	Human
F. De Lorenzi et al.^17^	2005	J Reconstr Microsurg.	Interrupted Micro-Mattress Sutures	Human
M. Akan et al.^18^	2006	Microsurgery	"Open Y″ Technique	Human
M. P. Suri et al.^19^	2009	J Reconstr Microsurg.	Vessel Reduction	Human
R. F. Rikard et al.^20^	2011	J Plast Reconstr Aesthet Surg.	Invaginating Anastomosis/Oblique End-To-End Anastomosis	Animal
T. Turker et al.^21^	2012	Hand Surg.	Branched Interpositional Vein Graft/The "Funnel" or "Trumpet" Graft	Human
R. Zen-Hu et al.^22^	2016	J Oral Maxillofac Surg.	Ren's Anastomoses	Human
A. Inbar et al.^23^	2019	J Reconstr Microsurg.	Modified Kunlin's Technique	Human
Y. Zhang et al.^24^	2020	Ann Plast Surg.	Mechanical Dilatation/Single-Mattress Suture/Wedge Resection	Human
U. Alamoudi et al.^25^	2021	Laryngoscope Invest Otolaryngol.	Vertical Arteriotomy	Human
X. Yang et al.^26^	2022	Br J Oral Maxillofac Surg.	Interposition Vein Graft	Human
A. Izadpanah^27^	2024	JPRAS Open.	Y-en-8 Anastomosis	/
L. Cheng et al.^28^	2024	J Orthop Surg Res.	Sucker-Like End-To-Side Arterial Anastomosis	Human

The various techniques could be grouped in either End-to-End Anastomosis variations, End-to-Side Anastomosis, use of Interposition Grafts, use of Microvascular Couplers.

Several specific techniques have been described to optimize End-to-End Anastomosis and the use of Interposition Grafts in cases of caliber mismatch.

The End-to-Side Anastomosis and the use of Microvascular Couplers,^45^ usually do not require special variations of the standard technique used for any vessels, even if variations of the end-to-side technique^28^ and coupler technique^46^ are reported.

**Fig. 1:**
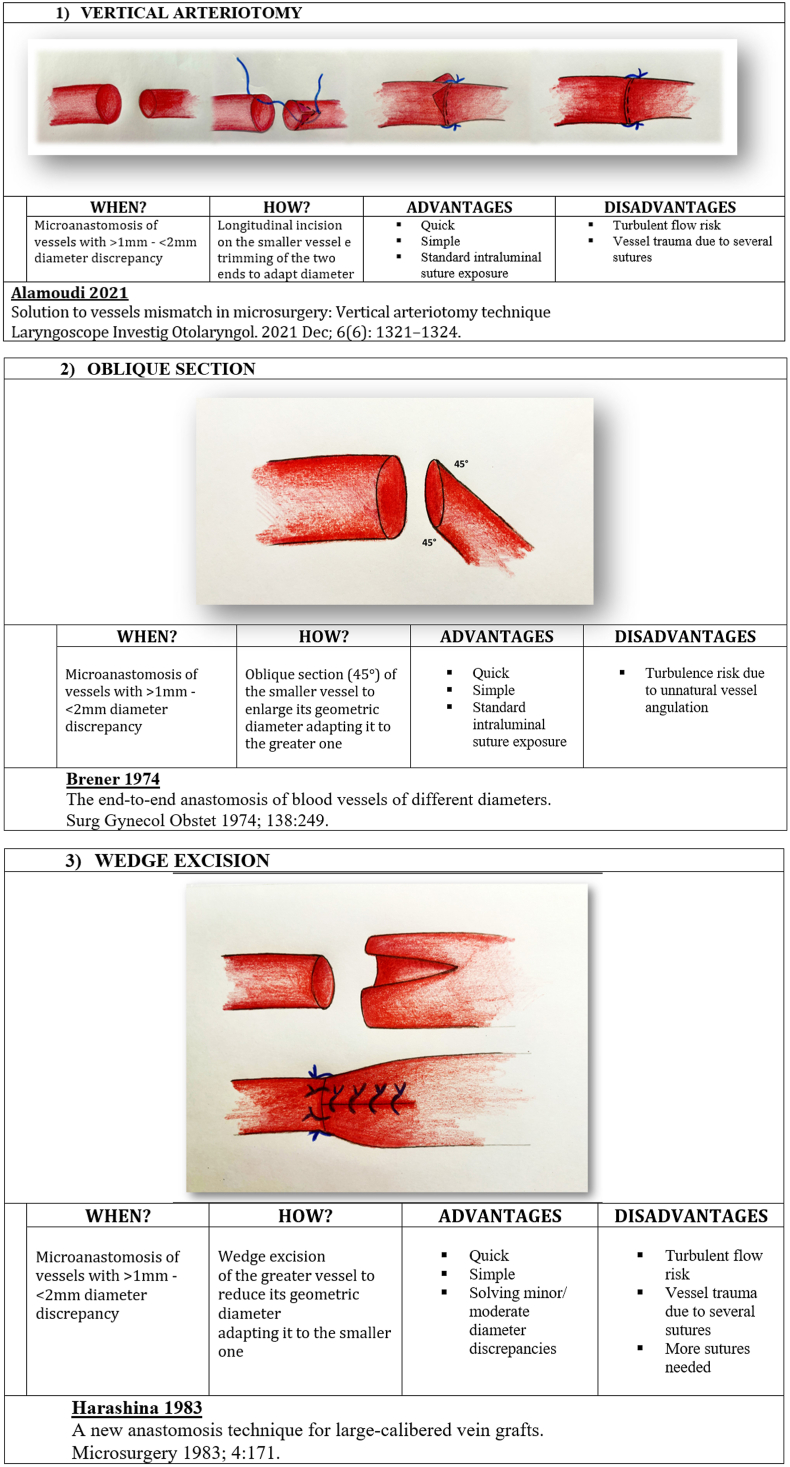
End-to-End Anastomosis variations techniques (details in the figure text).

**Fig. 2:**
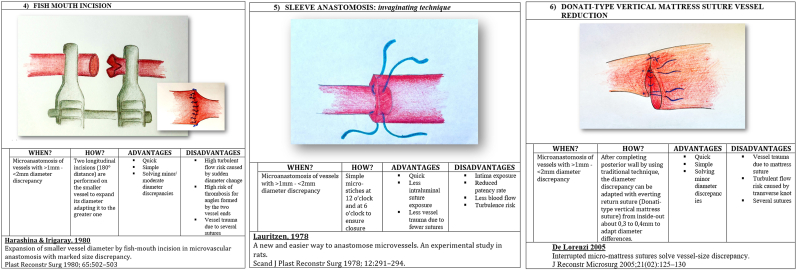
End-to-End Anastomosis variations techniques (details in the figure text).

**Fig. 3:**
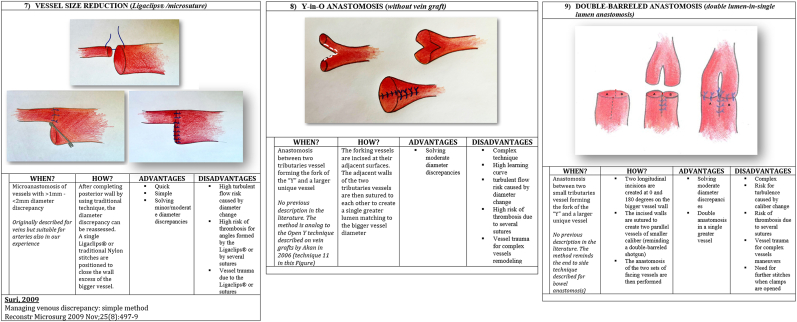
End-to-End Anastomosis variations techniques (details in the figure text).

**Fig. 4:**
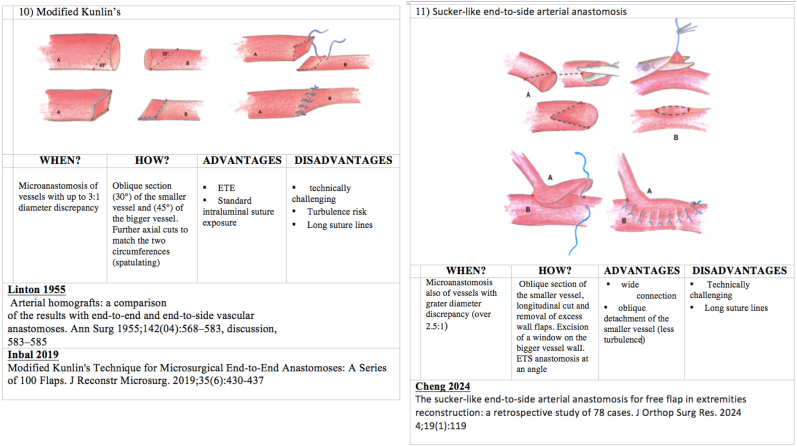
End-to-End and end to side Anastomosis variation techniques (details in the figure text).

**Fig. 5:**
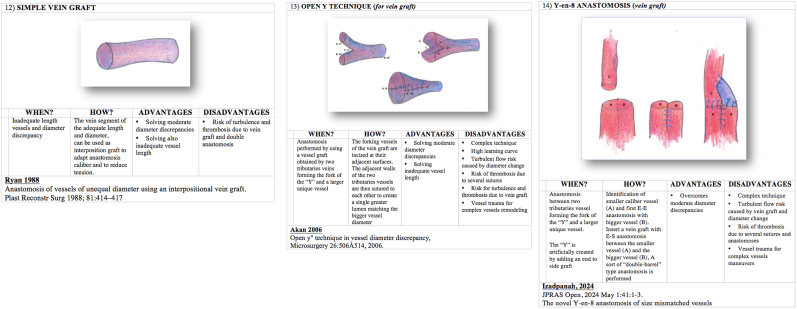
Techniques using interpositional grafts (details in the figure text).

We present in Fig. 1, Fig. 2, Fig. 3, Fig. 4 (Fig. 1, Fig. 2, Fig. 3, Fig. 4) the different techniques to manage caliber mismatch in microvascular anastomosis of the groups End-to-End Anastomosis variations (Fig. 1, Fig. 2, Fig. 3),^4^, ^5^, ^6^, ^7^^,^^17^^,^^19^^,^^23^^,^^25^ end to side (Fig. 4)^28^ and use of Interposition Grafts (Fig. 5)^8^^,^^18^^,^^27^ (twelve from the literature and two never described before). Indication, technique details, advantages and disadvantages of each technique are analyzed on the basis of the authors experience.

A. End-to-End Anastomosis variations techniques
1) Vertical Arteriotomy described by Alamoudi^25^ in 2021 requires a longitudinal incision in the smaller vessel. This enlarges the caliber, allowing a standard E-E anastomosis. Triangular vessel wall excess are then trimmed (Fig. 1).
2) Oblique Section. One of the oldest and simpler methods to increase the vessel end circumference was described in 1974^4^ (Fig. 1)
3) Wedge Excision^7^ technique obtains the reduction of the caliber of the larger vessel by removing a longitudinal wedge of its wall and suturing the defect (Fig. 1).
4) Fish Mouth Incision^6^ (Fig. 2). Two longitudinal incisions (180° distance) are performed on the smaller vessel to expand its diameter adapting it to the greater one
5) Sleeve Anastomosis: *Invaginating Technique*^5^ (Fig. 2). Suggested when the recipient vessel caliber is larger then the feedin vessel. Simple 2 or 3 micro-stiches maintain the smaller vessel inside the larger one allowing to float freely into it
6) Donati-Type Vertical Mattress Suture Vessel Reduction^17^ (Fig. 2).

After completing posterior wall by using traditional technique, we can adapt diameter discrepancy with return suture (Donati-type vertical mattress suture) from inside-out about 0,3 to 0,4 mm to adapt diameter differencies.

7) Vessel Size Reduction (*Ligaclip* ™*/Microsuture)* ^19.^ After completing posterior wall by using traditional technique, we can reassess exactly diameter discrepancy. We use a single Ligaclips® or traditional Nylon stitches to close excess of the lumen of the bigger vessel (Fig. 3).
8) Y-En-O Anastomosis (*Without Vein Graft).* The forking vessels are incised at their adjacent surfaces to create two opposite longitudinal section. The adiacent walls of the two tributaries vessels are then sutured to each other to create a single greater lumen adapting to the bigger vessel (Fig. 3).
9) Double-Barreled Anastomosis (*Double Lumens-En-Single Lumen Anastomosis).* After completing posterior wall by using traditional technique of the first vessel, we can perform the posterior wall also for the second vessel. Then we can assess exactly the anterior wall length that can be anastomised to the two separate small vessels (Fig. 3).
10) Modified Kunlin's Technique^23,29.^ An oblique section of the two vessels is performed. Further axial cuts are performed to match the two circumferences (Fig. 4).
B. End-to- side Anastomosis variation technique
11) Sucker-like end-to-side arterial anastomosis^28.^ After the oblique section of the smaller vessel, a longitudial cut and removal of excess wall flap is performed. On the bigger vesse, a window in the wall is created and an E-S anastomosis is performed at an angle (Fig. 4)
C Techniques using interpositional grafts
12) Simple Vein Graft.^8^ A vein graft of suitable caliber at the two ends is used to match the circumferences of the two vessels to be connected (Fig. 5).
13) Open Y Technique (*for vein graft)*.^18^ The forking vessels of the vein graft are incised at their adjacent surfaces to create two opposite longitudinal section. The adiacent walls of the two tributaries vessels are then sutured to each other to create a single greater lumen adapting to the bigger vessel. This vessel construct can be used as interposition graft (Fig. 5).
14) Y-En-8 Anastomosis (*vein graft)*^*27.*^ Identification of smaller caliber vessel (A) and first E-E anastomosis with bigger vessel (B); Insert a vein graft with E-S anastomosis between the smaller vessel (A) and the bigger vessel (B) (Fig. 5).

## 4 Discussion

The challenge of caliber mismatch in microvascular anastomosis remains a significant hurdle in reconstructive surgery and previous studies tried to summarize the available solutions.^30^

All the described technical variations may increase turbulence and the risk of thrombosis.^36^ The usual recommendations to optimize any microvascular anastomosis remain paramount: adequate choice of recipient vessels, basic principles of vessel handling before microsurgery, tension-free anastomosis, final preparation of vessels ends. These are discussed in detail in Table 2.

**Table 2:** The usual recommendations to optimize any microvascular anastomosis are discussed in detail.

Recommendation	You should aim for
**Adequate Choice of Recipient Vessels**	Healthy vessels
	Reasonable and adjustable size
	Good outflow
	Good position for anastomosis

**Basic Principles of Microsurgery**	Gentle dissection and minimal handling of vessel tissues
	Adequate debridement to remove damaged vessel
	Systematic and careful removal of the adventitia
	Relief of spasm: Mechanical dilatation
	Relief of spasm: Pharmacologic measures (Lidocaine or Papaverine)
	Keep the vessels hydrated

**Tension-Free Anastomosis**	Apply an adjustable approximating clamp to bring the vessel ends together for convenient suturing
	Avoid any kinking or twisting of the vessels distal to the anastomosis

**Final Preparation of Vessels Ends**	Resect sufficient adventitia
	Ensure right diameter and adequate length of the vessels before microanastomosis
	Apply correct tension to sutures
	Appropriate suture distancing

Since the published studies do not provide sufficient evidence on the best techniques to be used for microsurgical anastomosis of vessels of different calibers, this review synthesizes the various surgical techniques described to address this issue, highlighting their indications, efficacy, advantages and limitations.

The simplest method to overcome a modest difference in caliber is vessel dilation with dilators or forceps.^30^

If this method is not sufficient the next step consists in performing the end-to-end anastomosis using unequal bites trying to distribute the excess vessel wall circumference evenly.^11^ One of the risks is to have blood leakage between the sutures. Some authors suggest for better sealing (in caliber mismatch or in normal situations), to use laser, silicone cuffs or fibrin glue.^31^, ^32^, ^33^, ^34^

Cho and colleagues proved the efficacy and safety of glue to seal the gaps between sutures. Its use could, in our opinion, therefore be of aid in optimizing an end-to-end anastomosis with caliber mismatch.^35^

The discussion below evaluates the findings from the reviewed articles, comparing their approaches and outcomes.

### 4.1 End-to-end anastomosis (ETE)

End-to-end anastomosis is one of the most commonly used techniques for microvascular anastomosis. It is a simple and effective technique, especially when the diameter difference between vessels is minimal. If the mismatch is greater, technical variations can be used to adapt the diameter of the two vessels to be anastomosed, by increasing the caliber of the smaller vessel, or decreasing the one of the larger vessel. While these methods generally show high patency rates, they are not without complications. Issues such as increased tension at the anastomotic site and potential intimal damage can lead to thrombosis and stenosis.

Rickard and colleagues explain how the geometry of an anastomosis and shear stress gradients may influence its patency. With their computational study they demonstrate that, in theory and with several methodological limitations, when anastomosing smaller arteries to larger (1:2 ratio), between the four ETE techniques of Invaginating Anastomosis, Fish-Mouth Configuration, Oblique Configuration, Wedge Configuration, the latter is likely to cause least flow disturbance.^36^

In an experimental animal model study, they compared Invaginating technique and Oblique Configuration anastomosing smaller arteries to larger (1:1.5 to1:2.5 ratio). Oblique end-to-end technique was technically more difficult to perform with significantly higher revision rates. They therefore conclude that, in that model, the invagination technique (easier and faster) may be preferred.^37^

### 4.2 End-to-side anastomosis (ETS)

End-to-side anastomosis offers an alternative approach, particularly useful when there is a significant size discrepancy. This technique consists in connecting the smaller vessel to the side of the larger one, which can help in equalizing the flow dynamics and reducing the risk of tension-related complications. The technique has been extensively described and we did find only one technical variation to be used for caliber mismatch, the modified sucker-like ETS anastomosis technique that enlarges the connection by cutting obliquely the smaller vessel and anastomosing the arteries at a tilted angle.^28^

The ETS anastomosis has the advantage (paramount in lower limb reconstruction) of preserving the normal blood flow in the recipient vessel downstream from the anastomosis.

End-to-end and end to-side anastomosis patency rates have been extensively compared in experimental and clinical studies showing comparable results, with favorable outcomes, and in some studies, even lower rates of thrombosis and better long-term patency compared to end-to-end techniques.^38^, ^39^, ^40^, ^41^

However ETS anastomosis may require a slightly higher level of surgical skill and experience.

### 4.3 Interposition Grafts

The use of interposition vein (or rarely artery) grafts to bridge the size discrepancy can effectively address large size discrepancies and provide a versatile solution when direct anastomosis is not feasible. The primary drawback of this approach is the added complexity and potential for complications related to the graft itself, such as donor site morbidity and graft thrombosis. Although some studies prove that vein grafts don't increase the rate of thrombosis,^42^^,^^43^ others seem to show a higher failure risk.^10^^,^^44^

It is recommended, as technical tip, to position the vein graft according to the direction of the blood stream so that the venous valves don't obstruct the flow.^26^

### 4.4 Microvascular Couplers

Microvascular couplers are commonly used in routine venous anastomosis in free flap surgery.^45^ They have also gained popularity as a mechanical solution to the problem of caliber mismatch in veins.^46^ These devices simplify the venous anastomosis process and ensure consistent outcomes by standardizing the anastomotic technique. Studies indicate that couplers can achieve high patency rates and reduce operative time.^16^ However, their use is limited by the cost and the need for specialized equipment. Additionally, couplers, at the moment, are only suitable for veins, and not for extreme size discrepancies.

In Table 3 we synthesize our preferences of technique to be used to solve vessel caliber mismatch based on specific clinical scenarios.

**Table 3:** Tips and tricks for management of size discrepancies in microvascular anastomosis.

	SIMPLE END to END (E*to*E) MICROANASTOMOSIS	ADJUSTED END to END (E*to*E) MICROANASTOMOSIS	END to SIDE (E*to*S) MICROANASTOMOSIS
WHEN?	**SMALL** vessel size discrepancy	**MEDIUM** vessel size discrepancy >1 mm - <2 mm Or ≃30 %	**LARGE** vessel size discrepancy >2 mm Or >30 % - *Considerable size diameter discrepancy* - *Considerable wall thickness mismatch* - *To preserve downstream flow of the recipient vessels (lower limb)*
	<1 mm or <30 %		
	- *Small size diameter*		
	- *Vessel spasm*		
HOW?	⁃ Irrigate the lumen with solution of heparinized saline (1000 U/100 ml)		⁃ Irrigate the lumen with solution of heparinized saline (1000 U/100 ml) ⁃ Gentle dissection and adventitia removal ⁃ Place the recipient vessel in double clamps or haemostatic clamps (i.e. Satinski) for bigger vessels ⁃ Excise an adequate portion of lateral wall
	⁃ Gentle dilation		
	⁃ Topical Lidocaine (10 mg/mL) or Papaverine (3–4 mg/mL)		
	⁃ Evaluate vessels size discrepancy		
	⁃ Apply tips and tricks to match vessel lumen		
	⁃ Apply an adjustable approximating clamp to bring the vessel end together for convenient suturing		

SURGICAL METHOD	**Simple END TO END MICROANASTOMOSIS**	**Adjusted END TO END MICROANASTOMOSIS**	**END TO SIDE MICROANASTOMOSIS**
TIPS AND TRICKS		*1) Vertical Arteriotomy*	*11) Sucker-like end-to-side arterial anastomosis*
		*2) Oblique section*	
		*3) Wedge excision*	
		*4) Fish mouth incision*	
		*5) Sleeve anastomosis (invaginating)*	
		*6) Donati-type vertical mattress suture vessel reduction*	
		*7) Size vessel reduction (Ligaclip*^TM^*/microsuture)*	
		*8) Y-en-O anastomoses (without vein graft)*	
		*9) Double barreled anastomosis*	
		*10) Modified Kunlin's Technique*	
		*12) Simple vein graft*	
		*13) Open Y technique (for vein graft)*	
		*14) Y-en-8 anastomoses (vein graft)*	

When comparing the effectiveness of different techniques, it is clear that the choice of method depends largely on the specific clinical scenario, including the degree of size mismatch, the quality of the vessels, and the overall condition of the patient.

**Fig. 6:**
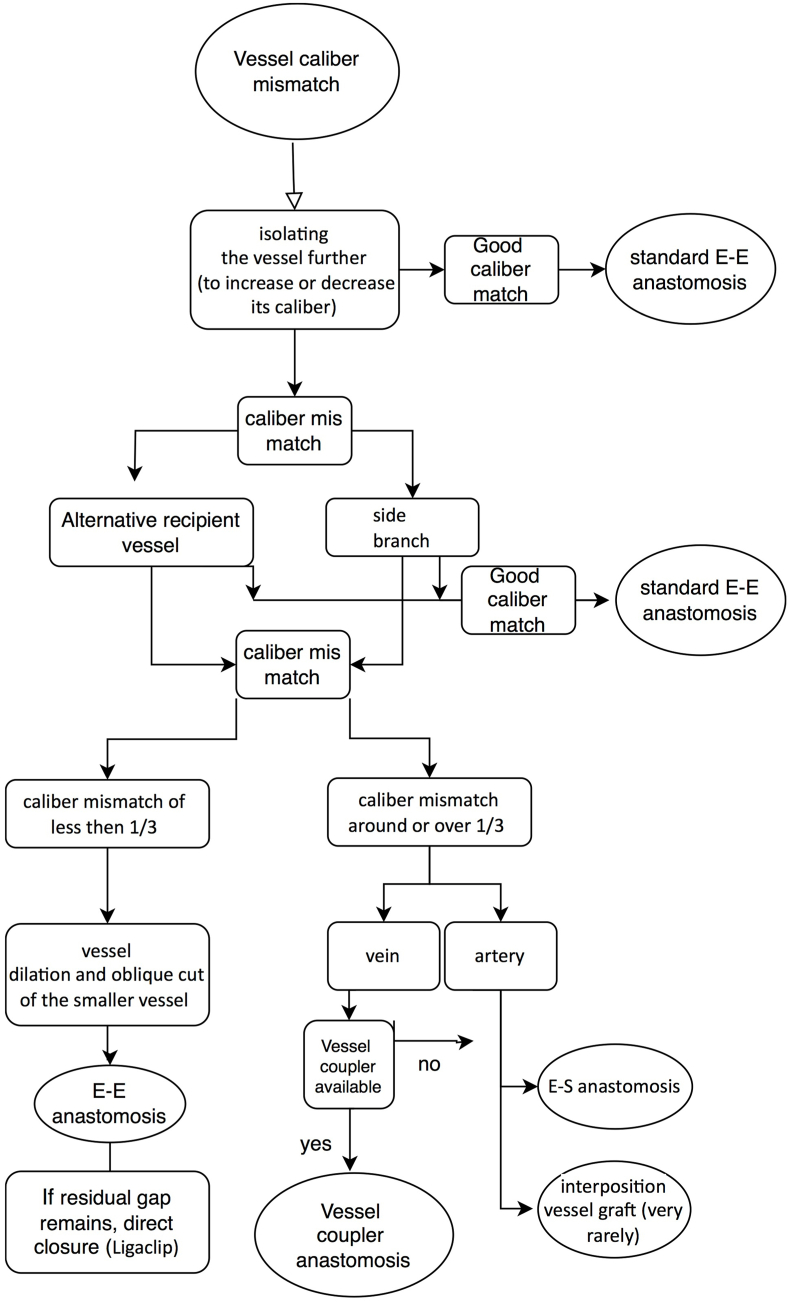
Flowchart on our preferred approach to a vessel mismatch to aid in the choice of the technique.

Our preferred approach to a vessel mismatch (Fig. 6) is to look for an alternative vessel. Either a new vessel or by isolating the vessel further to increase or decrease its caliber, or looking for a side branch to use to reduce caliber or to cut at the bifurcation to increase the caliber.

When these attempts do not work, for caliber mismatch of less then 1/3 our choice is for vessel dilation and oblique cut of the smaller vessel end. If, despite this attempt, at the end of the anastomosis a residual gap remains, direct closure of that excess vessel wall of the bigger vessel is performed, preferably with the positioning of a titanium small Ligaclip (LIGACLIP™, Ethicon US, LLC. 2023) in an oblique fashion to avoid a cul-de-sac.

A vessel coupler could be used for the vein, when available, usually for a mismatch up to ½ of the caliber if the quality of the vessel is adequate. If inadequate, the following techniques are preferred.

If caliber mismatch is around or over 1/3, we would prefer an end to side anastomosis.

The use of an interposition vessel graft, although elegant and easy, while requiring two end to end anastomosis, may increase the risk of thrombosis and is very rarely our preferred choice.

## 5 Limitations and Future directions

The studies reviewed have several limitations, including being experimental, preclinical or having small sample sizes, retrospective designs, and variability in reporting outcomes. Future research should focus on large-scale, multicenter, randomized controlled trials to provide more definitive evidence on the optimal techniques for managing caliber mismatch in microvascular anastomosis.

## 6 Conclusion

This literature review highlights the various surgical techniques employed to address caliber mismatch in microvascular anastomosis, each with its own set of advantages and limitations. While end-to-side anastomosis and interpositional grafts are effective for significant size discrepancies, end-to-end anastomosis and microvascular couplers offer reliable solutions for minor mismatches. Continued advancements in surgical technology and materials, coupled with rigorous clinical research, are essential to further refine these techniques and improve patient outcomes in microvascular surgery.

Fourteen surgical techniques to manage caliber mismatch in microvascular anastomosis (end to end and end to side variations and use of grafts) are depicted and explained. In the last two columns advantages and disadvantages of each.

1) Vertical Arteriotomy25
2) Oblique Section4
3) Wedge Excision7

4) Fish Mouth Incision6
5) Sleeve Anastomosis: Invaginating Technique5
6) Donati-Type Vertical Mattress Suture Vessel Reduction17

7) Vessel Size Reduction (Ligaclip TM/Microsuture) Sleeve Anastomosis: Invaginating Technique 19
8) Y-En-O Anastomosis (Without Vein Graft)
9) Double-Barreled Anastomosis (Double Lumens-En-Single Lumen Anastomosis)

10) Modified Kunlin's Technique23,29
11) Sucker-like end-to-side arterial anastomosis28

12) Simple Vein Graft8
13) Open Y Technique (for vein graft) 18
14) Y-En-8 Anastomosis (vein graft) 27

## Declaration of competing interest

The authors declare that they have no known competing financial interests or personal relationships that could have appeared to influence the work reported in this paper.
